# Bis{*N*′-[1-(2-pyrid­yl)ethyl­idene-κ*N*]benzohydrazidato-κ^2^ *N*′,*O*}nickel(II)

**DOI:** 10.1107/S1600536810007336

**Published:** 2010-03-03

**Authors:** Amitabha Datta, Nien-Tsu Chuang, Ming-Han Sie, Jui-Hsien Huang, Hon Man Lee

**Affiliations:** a Department of Chemistry, National Changhua University of Education, Changhua 50058, Taiwan

## Abstract

In the title complex, [Ni(C_14_H_12_N_3_O)_2_], the Ni^II^ atom lies at the centre of a distorted octahedron formed by two tridentate hydrazone ligands. Inter­molecular hydrogen bonds of the type C—H⋯*X* (*X* = N, O) link the complexes into a two-dimensional network.

## Related literature

For the preparation of the precursor ligand, see: Sen *et al.* (2005 ▶). For related complexes of the same ligand, see: Sen *et al.* (2005 ▶, 2007*a* ▶,*b* ▶), Ray *et al.* (2008 ▶).
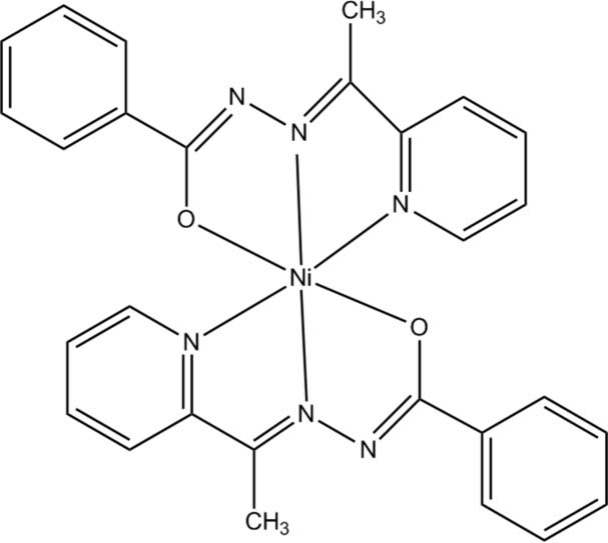


## Experimental

### 

#### Crystal data

[Ni(C_14_H_12_N_3_O)_2_]
*M* *_r_* = 535.24
Monoclinic, 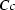

*a* = 10.248 (6) Å
*b* = 19.692 (11) Å
*c* = 12.281 (7) Å
β = 91.523 (10)°
*V* = 2477 (2) Å^3^
*Z* = 4
Mo *K*α radiation
μ = 0.82 mm^−1^
*T* = 298 K
0.37 × 0.33 × 0.25 mm

#### Data collection

Bruker SMART APEXII diffractometer
Absorption correction: multi-scan (*SADABS*; Sheldrick, 1996 ▶) *T*_min_ = 0.751, *T*_max_ = 0.821
5679 measured reflections
3600 independent reflections
3352 reflections with *I* > 2σ
*R*_int_ = 0.093

#### Refinement

*R*[*F* ^2^ > 2σ(*F* ^2^)] = 0.067
*wR*(*F* ^2^) = 0.182
*S* = 1.04
3600 reflections
336 parameters
2 restraints
H-atom parameters constrained
Δρ_max_ = 1.05 e Å^−3^
Δρ_min_ = −0.89 e Å^−3^
Absolute structure: Flack (1983 ▶), 1156 Friedel pairs
Flack parameter: 0.00 (2)

### 

Data collection: *APEX2* (Bruker, 2007 ▶); cell refinement: *SAINT* (Bruker, 2007 ▶); data reduction: *SAINT*; program(s) used to solve structure: *SHELXTL* (Sheldrick, 2008 ▶); program(s) used to refine structure: *SHELXTL*; molecular graphics: *SHELXTL*; software used to prepare material for publication: *DIAMOND* (Brandenburg, 1999 ▶).

## Supplementary Material

Crystal structure: contains datablocks I, global. DOI: 10.1107/S1600536810007336/jh2131sup1.cif

Structure factors: contains datablocks I. DOI: 10.1107/S1600536810007336/jh2131Isup2.hkl

Additional supplementary materials:  crystallographic information; 3D view; checkCIF report

## Figures and Tables

**Table 1:** Hydrogen-bond geometry (Å, °)

*D*—H⋯*A*	*D*—H	H⋯*A*	*D*⋯*A*	*D*—H⋯*A*
C21—H21*B*⋯N6	0.96	2.51	2.861 (10)	102
C10—H10⋯N3	0.93	2.51	2.813 (10)	100
C4—H4⋯O1^i^	0.93	2.51	3.164 (8)	128
C18—H18⋯O2^ii^	0.93	2.39	3.300 (9)	167

Symmetry codes: (i) 
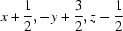
; (ii) 
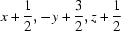
.

**Fig. 1.**
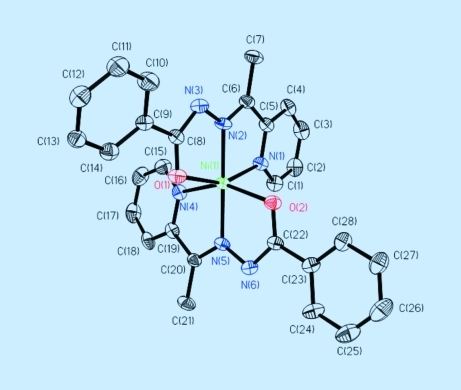
The structure of the title complex, showing 50% displacement ellipsoids for non-H atoms. H atoms are excluded for clarity.

**Fig. 2.**
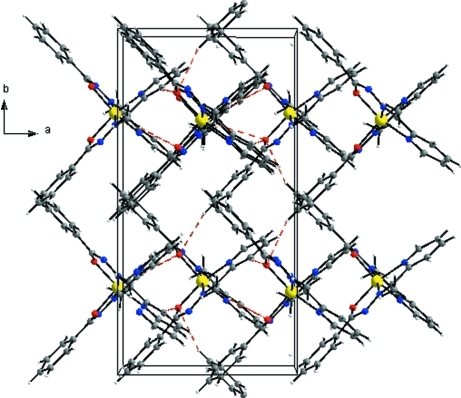
A packing diagram of the title compound along the c-axis showing the intermolecular hydrogen bonds (dashed lines).

## supplementary crystallographic information

### Comment

The title complex adopts a distorted octahedron geometry with two tridentate hydrazone ligands. While the N–Ni–N angle is 174.5 (2)°, which is close to the ideal 180°, the two O–Ni–N angles are much smaller (154.7 (2)° and 153.9 (2)°).

An intramolecular non-classical hydrogen bond of the type C—H···N is present. Non-classical intermolecular hydrogen bonds of type C—H···N and C—H···O also link complexes into a two-dimensional network.

Copper (Sen *et al.* 2007a)(Sen *et al.* 2007b), cadmium (Sen *et al.* 2005), zinc (Ray *et al.* 2008) and manganese (Ray *et al.* 2008) complexes of the same ligand have been published.

### Experimental

The ligand precursor, [C_6_H_5_C(O)NHN=C(CH_3_)C_5_H_4_N] (LH) was prepared according to a literature procedure (Sen *et al.* 2005). To a methanolic solution (20 ml) of nickel chloride hexahydrate (0.237 g, 1.0 mmol), LH (0.478 g, 2 mmol) was added and then kept at room temperature. After a few days, dark brown, rectangular crystals of the title compound suitable for X-ray diffraction studies were formed. Crystals were collected and dried in the air. Yield: 0.147 g, 62%.

### Refinement

All the H atoms were positioned geometrically and refined as riding atoms, with C_aryl_—H = 0.93, C_methyl_—H = 0.96, Å while *U_i_*_so_(H) = 1.5 *U_eq_* (C) for the methyl H atoms and 1.2 *U_eq_* (C) for all the other H atoms.

### Crystal data

**Table:**

[Ni(C_14_H_12_N_3_O)_2_]	*F*(000) = 1112
*M**_r_* = 535.24	*D*_x_ = 1.435 Mg m^−^^3^
Monoclinic, *C**c*	Mo *K*α radiation, λ = 0.71073 Å
Hall symbol: C -2yc	Cell parameters from 3920 reflections
*a* = 10.248 (6) Å	θ = 2.2–26.3°
*b* = 19.692 (11) Å	µ = 0.82 mm^−^^1^
*c* = 12.281 (7) Å	*T* = 298 K
β = 91.523 (10)°	Parallelepiped, brown
*V* = 2477 (2) Å^3^	0.37 × 0.33 × 0.25 mm
*Z* = 4	

### Data collection

**Table:**

Bruker SMART APEXII diffractometer	3600 independent reflections
Radiation source: fine-focus sealed tube	3352 reflections with *I* > 2σ
graphite	*R*_int_ = 0.093
ω scans	θ_max_ = 26.3°, θ_min_ = 2.1°
Absorption correction: multi-scan (*SADABS*; Sheldrick, 1996)	*h* = −12→10
*T*_min_ = 0.751, *T*_max_ = 0.821	*k* = −22→24
5679 measured reflections	*l* = −15→13

### Refinement

**Table:**

Refinement on *F*^2^	Secondary atom site location: difference Fourier map
Least-squares matrix: full	Hydrogen site location: inferred from neighbouring sites
*R*[*F*^2^ > 2σ(*F*^2^)] = 0.067	H-atom parameters constrained
*wR*(*F*^2^) = 0.182	*w* = 1/[σ^2^(*F*_o_^2^) + (0.1528*P*)^2^] where *P* = (*F*_o_^2^ + 2*F*_c_^2^)/3
*S* = 1.04	(Δ/σ)_max_ = 0.001
3600 reflections	Δρ_max_ = 1.05 e Å^−^^3^
336 parameters	Δρ_min_ = −0.88 e Å^−^^3^
2 restraints	Absolute structure: Flack (1983), 1156 Friedel pairs
Primary atom site location: structure-invariant direct methods	Flack parameter: 0.00 (2)

### Special details

**Table:**

Geometry. All e.s.d.'s (except the e.s.d. in the dihedral angle between two l.s. planes) are estimated using the full covariance matrix. The cell e.s.d.'s are taken into account individually in the estimation of e.s.d.'s in distances, angles and torsion angles; correlations between e.s.d.'s in cell parameters are only used when they are defined by crystal symmetry. An approximate (isotropic) treatment of cell e.s.d.'s is used for estimating e.s.d.'s involving l.s. planes.
Refinement. Refinement of *F*^2^ against ALL reflections. The weighted *R*-factor wR and goodness of fit *S* are based on *F*^2^, conventional *R*-factors *R* are based on *F*, with *F* set to zero for negative *F*^2^. The threshold expression of *F*^2^ > σ(*F*^2^) is used only for calculating *R*-factors(gt) etc. and is not relevant to the choice of reflections for refinement. *R*-factors based on *F*^2^ are statistically about twice as large as those based on *F*, and *R*- factors based on ALL data will be even larger.

### Fractional atomic coordinates and isotropic or equivalent isotropic displacement parameters (Å^2^)

**Table:**

	*x*	*y*	*z*	*U*_iso_*/*U*_eq_	
C1	1.1923 (7)	0.6618 (3)	0.3808 (6)	0.0451 (14)	
H1	1.1942	0.6524	0.4550	0.054*	
C2	1.2836 (8)	0.6300 (4)	0.3159 (7)	0.0561 (18)	
H2	1.3456	0.6005	0.3460	0.067*	
C3	1.2791 (7)	0.6435 (4)	0.2071 (7)	0.0552 (18)	
H3	1.3385	0.6229	0.1616	0.066*	
C4	1.1860 (7)	0.6880 (4)	0.1640 (6)	0.0467 (15)	
H4	1.1812	0.6968	0.0896	0.056*	
C5	1.0994 (6)	0.7192 (3)	0.2350 (5)	0.0370 (12)	
C6	1.0010 (7)	0.7708 (3)	0.1986 (5)	0.0404 (14)	
C7	0.9737 (8)	0.7881 (4)	0.0812 (5)	0.0532 (17)	
H7A	0.9677	0.8365	0.0731	0.080*	
H7B	0.8929	0.7676	0.0575	0.080*	
H7C	1.0432	0.7712	0.0378	0.080*	
C8	0.8049 (6)	0.8668 (3)	0.3545 (5)	0.0364 (12)	
C9	0.7033 (7)	0.9222 (3)	0.3491 (5)	0.0400 (13)	
C10	0.6377 (9)	0.9389 (4)	0.2532 (7)	0.064 (2)	
H10	0.6593	0.9167	0.1893	0.076*	
C11	0.5411 (9)	0.9875 (4)	0.2496 (7)	0.067 (2)	
H11	0.4971	0.9973	0.1843	0.080*	
C12	0.5094 (8)	1.0221 (4)	0.3453 (7)	0.0556 (18)	
H12	0.4448	1.0553	0.3439	0.067*	
C13	0.5742 (8)	1.0065 (3)	0.4398 (6)	0.0487 (16)	
H13	0.5536	1.0294	0.5033	0.058*	
C14	0.6709 (7)	0.9568 (3)	0.4436 (6)	0.0473 (15)	
H14	0.7138	0.9466	0.5093	0.057*	
C15	1.2059 (8)	0.8590 (4)	0.4491 (6)	0.0527 (17)	
H15	1.1929	0.8680	0.3752	0.063*	
C16	1.3118 (9)	0.8881 (4)	0.5021 (8)	0.063 (2)	
H16	1.3707	0.9146	0.4646	0.076*	
C17	1.3282 (8)	0.8767 (4)	0.6122 (8)	0.064 (2)	
H17	1.3977	0.8967	0.6504	0.076*	
C18	1.2418 (7)	0.8358 (3)	0.6658 (6)	0.0495 (16)	
H18	1.2515	0.8283	0.7403	0.059*	
C19	1.1398 (6)	0.8059 (3)	0.6063 (5)	0.0360 (12)	
C20	1.0465 (7)	0.7564 (3)	0.6536 (5)	0.0374 (14)	
C21	1.0476 (10)	0.7429 (4)	0.7719 (6)	0.0523 (19)	
H21A	1.1219	0.7154	0.7914	0.078*	
H21B	0.9691	0.7196	0.7903	0.078*	
H21C	1.0528	0.7852	0.8108	0.078*	
C22	0.8200 (6)	0.6570 (3)	0.5195 (5)	0.0358 (12)	
C23	0.7290 (6)	0.6000 (3)	0.5351 (5)	0.0384 (12)	
C24	0.7114 (8)	0.5723 (4)	0.6395 (6)	0.0568 (18)	
H24	0.7605	0.5879	0.6992	0.068*	
C25	0.6194 (10)	0.5212 (5)	0.6517 (9)	0.081 (3)	
H25	0.6088	0.5021	0.7202	0.098*	
C26	0.5425 (10)	0.4977 (5)	0.5641 (9)	0.075 (3)	
H26	0.4785	0.4650	0.5743	0.090*	
C27	0.5632 (8)	0.5235 (4)	0.4637 (8)	0.061 (2)	
H27	0.5155	0.5067	0.4042	0.073*	
C28	0.6546 (7)	0.5747 (3)	0.4480 (6)	0.0462 (14)	
H28	0.6659	0.5921	0.3785	0.055*	
N1	1.1029 (5)	0.7048 (3)	0.3422 (4)	0.0379 (11)	
N2	0.9417 (5)	0.7980 (3)	0.2778 (4)	0.0338 (10)	
N3	0.8495 (6)	0.8480 (3)	0.2592 (4)	0.0414 (11)	
N4	1.1212 (5)	0.8188 (3)	0.4977 (4)	0.0389 (11)	
N5	0.9746 (6)	0.7265 (2)	0.5806 (4)	0.0352 (11)	
N6	0.8911 (5)	0.6760 (3)	0.6080 (4)	0.0373 (11)	
Ni1	0.96750 (6)	0.76219 (3)	0.42846 (5)	0.0314 (2)	
O1	0.8347 (5)	0.8422 (2)	0.4467 (4)	0.0439 (10)	
O2	0.8288 (5)	0.6838 (2)	0.4245 (4)	0.0415 (10)	

### Atomic displacement parameters (Å^2^)

**Table:**

	*U*^11^	*U*^22^	*U*^33^	*U*^12^	*U*^13^	*U*^23^
C1	0.027 (3)	0.057 (3)	0.052 (4)	0.004 (3)	0.000 (3)	−0.001 (3)
C2	0.035 (4)	0.057 (4)	0.077 (6)	0.008 (3)	0.002 (3)	−0.007 (3)
C3	0.035 (4)	0.065 (4)	0.067 (5)	0.005 (3)	0.016 (3)	−0.016 (3)
C4	0.033 (4)	0.062 (4)	0.045 (4)	−0.004 (3)	0.012 (3)	−0.010 (3)
C5	0.027 (3)	0.049 (3)	0.035 (3)	−0.009 (3)	0.006 (2)	−0.004 (2)
C6	0.041 (4)	0.050 (3)	0.031 (3)	−0.012 (3)	0.007 (3)	0.001 (2)
C7	0.057 (5)	0.072 (4)	0.030 (3)	−0.007 (4)	0.004 (3)	0.005 (3)
C8	0.027 (3)	0.044 (3)	0.038 (3)	0.000 (2)	0.002 (2)	0.000 (2)
C9	0.035 (3)	0.047 (3)	0.038 (3)	−0.001 (3)	0.001 (2)	0.004 (2)
C10	0.068 (6)	0.068 (4)	0.055 (4)	0.027 (4)	−0.009 (4)	0.000 (3)
C11	0.070 (6)	0.067 (4)	0.062 (5)	0.031 (4)	−0.019 (4)	0.002 (4)
C12	0.042 (4)	0.057 (4)	0.069 (5)	0.010 (3)	0.006 (3)	0.009 (3)
C13	0.051 (4)	0.051 (3)	0.045 (4)	0.001 (3)	0.010 (3)	0.002 (3)
C14	0.042 (4)	0.053 (3)	0.047 (4)	−0.002 (3)	0.003 (3)	0.007 (3)
C15	0.052 (5)	0.057 (4)	0.049 (4)	−0.010 (3)	−0.006 (3)	0.006 (3)
C16	0.050 (5)	0.055 (4)	0.085 (6)	−0.018 (4)	−0.001 (4)	0.004 (4)
C17	0.046 (5)	0.057 (4)	0.086 (6)	−0.008 (3)	−0.023 (4)	−0.011 (4)
C18	0.045 (4)	0.050 (3)	0.053 (4)	0.005 (3)	−0.013 (3)	−0.017 (3)
C19	0.022 (3)	0.042 (3)	0.044 (3)	0.011 (2)	−0.003 (2)	−0.005 (2)
C20	0.039 (4)	0.042 (3)	0.031 (3)	0.007 (2)	−0.002 (3)	−0.003 (2)
C21	0.058 (6)	0.065 (4)	0.033 (4)	0.012 (3)	−0.009 (3)	0.000 (3)
C22	0.029 (3)	0.047 (3)	0.031 (3)	0.005 (2)	0.004 (2)	−0.002 (2)
C23	0.021 (3)	0.049 (3)	0.046 (3)	0.000 (2)	0.009 (2)	0.005 (2)
C24	0.054 (5)	0.066 (4)	0.051 (4)	−0.001 (4)	0.009 (3)	0.021 (3)
C25	0.079 (7)	0.088 (6)	0.079 (7)	−0.016 (5)	0.025 (6)	0.028 (5)
C26	0.056 (5)	0.067 (5)	0.103 (8)	−0.014 (4)	0.018 (5)	0.017 (5)
C27	0.044 (4)	0.056 (4)	0.083 (6)	−0.012 (3)	0.006 (4)	0.004 (4)
C28	0.031 (3)	0.051 (3)	0.057 (4)	−0.007 (3)	0.002 (3)	−0.001 (3)
N1	0.026 (3)	0.049 (3)	0.039 (3)	−0.004 (2)	0.002 (2)	−0.001 (2)
N2	0.021 (2)	0.053 (3)	0.027 (2)	−0.0020 (19)	0.0042 (17)	0.0075 (19)
N3	0.036 (3)	0.052 (3)	0.036 (3)	0.004 (2)	0.002 (2)	0.009 (2)
N4	0.024 (2)	0.046 (3)	0.047 (3)	−0.004 (2)	−0.002 (2)	−0.003 (2)
N5	0.030 (3)	0.048 (2)	0.028 (3)	0.008 (2)	0.002 (2)	−0.0004 (18)
N6	0.030 (3)	0.046 (2)	0.037 (3)	0.001 (2)	0.001 (2)	0.0061 (19)
Ni1	0.0219 (4)	0.0438 (3)	0.0284 (4)	−0.0005 (3)	0.0006 (2)	0.0020 (3)
O1	0.039 (3)	0.057 (2)	0.036 (2)	0.010 (2)	0.0046 (19)	0.0086 (18)
O2	0.036 (3)	0.056 (2)	0.032 (2)	−0.0078 (19)	−0.0022 (18)	0.0039 (17)

### Geometric parameters (Å, °)

**Table:**

C1—N1	1.326 (9)	C16—H16	0.9300
C1—C2	1.393 (10)	C17—C18	1.378 (12)
C1—H1	0.9300	C17—H17	0.9300
C2—C3	1.362 (12)	C18—C19	1.389 (9)
C2—H2	0.9300	C18—H18	0.9300
C3—C4	1.391 (12)	C19—N4	1.366 (8)
C3—H3	0.9300	C19—C20	1.493 (9)
C4—C5	1.402 (9)	C20—N5	1.288 (9)
C4—H4	0.9300	C20—C21	1.477 (10)
C5—N1	1.346 (8)	C21—H21A	0.9600
C5—C6	1.492 (10)	C21—H21B	0.9600
C6—N2	1.277 (9)	C21—H21C	0.9600
C6—C7	1.501 (9)	C22—O2	1.285 (8)
C7—H7A	0.9600	C22—N6	1.346 (8)
C7—H7B	0.9600	C22—C23	1.476 (9)
C7—H7C	0.9600	C23—C28	1.389 (10)
C8—O1	1.262 (8)	C23—C24	1.410 (9)
C8—N3	1.321 (8)	C24—C25	1.390 (12)
C8—C9	1.508 (9)	C24—H24	0.9300
C9—C10	1.381 (10)	C25—C26	1.396 (16)
C9—C14	1.393 (10)	C25—H25	0.9300
C10—C11	1.376 (11)	C26—C27	1.355 (13)
C10—H10	0.9300	C26—H26	0.9300
C11—C12	1.404 (12)	C27—C28	1.394 (10)
C11—H11	0.9300	C27—H27	0.9300
C12—C13	1.358 (11)	C28—H28	0.9300
C12—H12	0.9300	N1—Ni1	2.099 (5)
C13—C14	1.393 (10)	N2—N3	1.380 (8)
C13—H13	0.9300	N2—Ni1	1.992 (5)
C14—H14	0.9300	N4—Ni1	2.091 (5)
C15—N4	1.329 (9)	N5—N6	1.359 (8)
C15—C16	1.375 (12)	N5—Ni1	1.995 (5)
C15—H15	0.9300	Ni1—O1	2.098 (5)
C16—C17	1.376 (12)	Ni1—O2	2.098 (5)
			
N1—C1—C2	123.4 (7)	N5—C20—C19	112.9 (6)
N1—C1—H1	118.3	C21—C20—C19	120.7 (6)
C2—C1—H1	118.3	C20—C21—H21A	109.5
C3—C2—C1	117.9 (7)	C20—C21—H21B	109.5
C3—C2—H2	121.1	H21A—C21—H21B	109.5
C1—C2—H2	121.1	C20—C21—H21C	109.5
C2—C3—C4	120.1 (6)	H21A—C21—H21C	109.5
C2—C3—H3	120.0	H21B—C21—H21C	109.5
C4—C3—H3	120.0	O2—C22—N6	124.7 (6)
C3—C4—C5	118.6 (7)	O2—C22—C23	119.3 (5)
C3—C4—H4	120.7	N6—C22—C23	116.0 (5)
C5—C4—H4	120.7	C28—C23—C24	118.7 (7)
N1—C5—C4	121.0 (6)	C28—C23—C22	120.6 (6)
N1—C5—C6	115.9 (5)	C24—C23—C22	120.6 (6)
C4—C5—C6	123.1 (6)	C25—C24—C23	118.8 (9)
N2—C6—C5	112.9 (6)	C25—C24—H24	120.6
N2—C6—C7	123.8 (7)	C23—C24—H24	120.6
C5—C6—C7	123.3 (6)	C24—C25—C26	121.8 (8)
C6—C7—H7A	109.5	C24—C25—H25	119.1
C6—C7—H7B	109.5	C26—C25—H25	119.1
H7A—C7—H7B	109.5	C27—C26—C25	118.6 (8)
C6—C7—H7C	109.5	C27—C26—H26	120.7
H7A—C7—H7C	109.5	C25—C26—H26	120.7
H7B—C7—H7C	109.5	C26—C27—C28	121.3 (9)
O1—C8—N3	127.4 (6)	C26—C27—H27	119.3
O1—C8—C9	117.8 (5)	C28—C27—H27	119.3
N3—C8—C9	114.7 (5)	C23—C28—C27	120.7 (7)
C10—C9—C14	118.2 (7)	C23—C28—H28	119.7
C10—C9—C8	122.0 (6)	C27—C28—H28	119.7
C14—C9—C8	119.8 (6)	C1—N1—C5	119.0 (6)
C11—C10—C9	121.8 (8)	C1—N1—Ni1	128.6 (5)
C11—C10—H10	119.1	C5—N1—Ni1	112.3 (4)
C9—C10—H10	119.1	C6—N2—N3	120.7 (5)
C10—C11—C12	119.5 (7)	C6—N2—Ni1	120.3 (5)
C10—C11—H11	120.2	N3—N2—Ni1	118.7 (4)
C12—C11—H11	120.2	C8—N3—N2	107.7 (5)
C13—C12—C11	119.2 (7)	C15—N4—C19	118.3 (6)
C13—C12—H12	120.4	C15—N4—Ni1	129.0 (5)
C11—C12—H12	120.4	C19—N4—Ni1	112.5 (4)
C12—C13—C14	121.3 (7)	C20—N5—N6	120.9 (5)
C12—C13—H13	119.4	C20—N5—Ni1	119.7 (5)
C14—C13—H13	119.4	N6—N5—Ni1	118.8 (4)
C13—C14—C9	120.1 (7)	C22—N6—N5	109.5 (5)
C13—C14—H14	120.0	N2—Ni1—N5	174.5 (2)
C9—C14—H14	120.0	N2—Ni1—N4	105.6 (2)
N4—C15—C16	123.5 (7)	N5—Ni1—N4	78.4 (2)
N4—C15—H15	118.3	N2—Ni1—O1	76.29 (19)
C16—C15—H15	118.3	N5—Ni1—O1	99.93 (19)
C15—C16—C17	118.2 (7)	N4—Ni1—O1	92.3 (2)
C15—C16—H16	120.9	N2—Ni1—O2	99.56 (19)
C17—C16—H16	120.9	N5—Ni1—O2	76.7 (2)
C16—C17—C18	120.1 (7)	N4—Ni1—O2	154.7 (2)
C16—C17—H17	120.0	O1—Ni1—O2	96.6 (2)
C18—C17—H17	120.0	N2—Ni1—N1	78.2 (2)
C17—C18—C19	118.7 (7)	N5—Ni1—N1	105.9 (2)
C17—C18—H18	120.6	N4—Ni1—N1	89.5 (2)
C19—C18—H18	120.6	O1—Ni1—N1	153.90 (19)
N4—C19—C18	121.2 (6)	O2—Ni1—N1	92.8 (2)
N4—C19—C20	115.2 (5)	C8—O1—Ni1	109.5 (4)
C18—C19—C20	123.6 (6)	C22—O2—Ni1	110.1 (4)
N5—C20—C21	126.3 (7)		
			
N1—C1—C2—C3	−0.8 (11)	C18—C19—N4—Ni1	176.5 (5)
C1—C2—C3—C4	0.3 (11)	C20—C19—N4—Ni1	−1.5 (6)
C2—C3—C4—C5	1.2 (11)	C21—C20—N5—N6	−1.5 (10)
C3—C4—C5—N1	−2.3 (10)	C19—C20—N5—N6	174.9 (5)
C3—C4—C5—C6	176.2 (6)	C21—C20—N5—Ni1	170.3 (6)
N1—C5—C6—N2	5.6 (8)	C19—C20—N5—Ni1	−13.3 (7)
C4—C5—C6—N2	−173.0 (6)	O2—C22—N6—N5	−1.3 (8)
N1—C5—C6—C7	−173.6 (6)	C23—C22—N6—N5	177.0 (5)
C4—C5—C6—C7	7.8 (10)	C20—N5—N6—C22	175.0 (6)
O1—C8—C9—C10	−161.4 (7)	Ni1—N5—N6—C22	3.1 (6)
N3—C8—C9—C10	16.3 (10)	C6—N2—Ni1—N4	92.0 (5)
O1—C8—C9—C14	16.8 (9)	N3—N2—Ni1—N4	−94.3 (4)
N3—C8—C9—C14	−165.5 (6)	C6—N2—Ni1—O1	−179.5 (5)
C14—C9—C10—C11	−1.0 (13)	N3—N2—Ni1—O1	−5.7 (4)
C8—C9—C10—C11	177.2 (8)	C6—N2—Ni1—O2	−85.0 (5)
C9—C10—C11—C12	1.2 (14)	N3—N2—Ni1—O2	88.7 (4)
C10—C11—C12—C13	−0.5 (13)	C6—N2—Ni1—N1	5.9 (5)
C11—C12—C13—C14	−0.2 (12)	N3—N2—Ni1—N1	179.6 (5)
C12—C13—C14—C9	0.3 (11)	C20—N5—Ni1—N4	10.0 (5)
C10—C9—C14—C13	0.3 (10)	N6—N5—Ni1—N4	−178.1 (4)
C8—C9—C14—C13	−178.0 (6)	C20—N5—Ni1—O1	−80.3 (5)
N4—C15—C16—C17	−2.3 (13)	N6—N5—Ni1—O1	91.6 (4)
C15—C16—C17—C18	1.7 (13)	C20—N5—Ni1—O2	−174.8 (5)
C16—C17—C18—C19	0.8 (11)	N6—N5—Ni1—O2	−2.8 (4)
C17—C18—C19—N4	−2.8 (9)	C20—N5—Ni1—N1	96.1 (5)
C17—C18—C19—C20	175.0 (6)	N6—N5—Ni1—N1	−91.9 (4)
N4—C19—C20—N5	9.3 (7)	C15—N4—Ni1—N2	−14.2 (7)
C18—C19—C20—N5	−168.7 (6)	C19—N4—Ni1—N2	172.2 (4)
N4—C19—C20—C21	−174.1 (6)	C15—N4—Ni1—N5	169.7 (7)
C18—C19—C20—C21	8.0 (9)	C19—N4—Ni1—N5	−3.8 (4)
O2—C22—C23—C28	0.9 (9)	C15—N4—Ni1—O1	−90.6 (6)
N6—C22—C23—C28	−177.6 (6)	C19—N4—Ni1—O1	95.8 (4)
O2—C22—C23—C24	−176.2 (6)	C15—N4—Ni1—O2	158.8 (6)
N6—C22—C23—C24	5.4 (9)	C19—N4—Ni1—O2	−14.7 (7)
C28—C23—C24—C25	−0.6 (11)	C15—N4—Ni1—N1	63.3 (6)
C22—C23—C24—C25	176.5 (7)	C19—N4—Ni1—N1	−110.2 (4)
C23—C24—C25—C26	−1.3 (14)	C1—N1—Ni1—N2	173.1 (6)
C24—C25—C26—C27	3.2 (16)	C5—N1—Ni1—N2	−2.1 (4)
C25—C26—C27—C28	−3.1 (14)	C1—N1—Ni1—N5	−10.8 (6)
C24—C23—C28—C27	0.8 (10)	C5—N1—Ni1—N5	174.0 (4)
C22—C23—C28—C27	−176.3 (6)	C1—N1—Ni1—N4	67.0 (6)
C26—C27—C28—C23	1.1 (12)	C5—N1—Ni1—N4	−108.2 (4)
C2—C1—N1—C5	−0.3 (10)	C1—N1—Ni1—O1	161.2 (5)
C2—C1—N1—Ni1	−175.2 (5)	C5—N1—Ni1—O1	−14.0 (7)
C4—C5—N1—C1	1.8 (9)	C1—N1—Ni1—O2	−87.8 (6)
C6—C5—N1—C1	−176.8 (6)	C5—N1—Ni1—O2	97.0 (4)
C4—C5—N1—Ni1	177.6 (5)	N3—C8—O1—Ni1	−1.3 (8)
C6—C5—N1—Ni1	−1.0 (6)	C9—C8—O1—Ni1	176.1 (4)
C5—C6—N2—N3	178.5 (5)	N2—Ni1—O1—C8	3.6 (4)
C7—C6—N2—N3	−2.3 (10)	N5—Ni1—O1—C8	−172.2 (4)
C5—C6—N2—Ni1	−7.9 (7)	N4—Ni1—O1—C8	109.1 (4)
C7—C6—N2—Ni1	171.3 (5)	O2—Ni1—O1—C8	−94.6 (4)
O1—C8—N3—N2	−3.2 (9)	N1—Ni1—O1—C8	15.6 (7)
C9—C8—N3—N2	179.4 (5)	N6—C22—O2—Ni1	−0.9 (7)
C6—N2—N3—C8	−179.8 (6)	C23—C22—O2—Ni1	−179.2 (4)
Ni1—N2—N3—C8	6.5 (7)	N2—Ni1—O2—C22	−173.9 (4)
C16—C15—N4—C19	0.4 (11)	N5—Ni1—O2—C22	1.9 (4)
C16—C15—N4—Ni1	−172.8 (6)	N4—Ni1—O2—C22	12.9 (7)
C18—C19—N4—C15	2.2 (9)	O1—Ni1—O2—C22	−96.8 (4)
C20—C19—N4—C15	−175.8 (6)	N1—Ni1—O2—C22	107.6 (4)

### Hydrogen-bond geometry (Å, °)

**Table:**

*D*—H···*A*	*D*—H	H···*A*	*D*···*A*	*D*—H···*A*
C21—H21B···N6	0.96	2.51	2.861 (10)	102
C10—H10···N3	0.93	2.51	2.813 (10)	100
C4—H4···O1^i^	0.93	2.51	3.164 (8)	128
C18—H18···O2^ii^	0.93	2.39	3.300 (9)	167

Symmetry codes: (i) *x*+1/2, −*y*+3/2, *z*−1/2; (ii) *x*+1/2, −*y*+3/2, *z*+1/2.
